# Female Soccer Players’ Psychological Profile: Differences between Professional and Amateur Players

**DOI:** 10.3390/ijerph17124357

**Published:** 2020-06-18

**Authors:** Cecilia Ruiz-Esteban, Aurelio Olmedilla, Inmaculada Méndez, Juan Jesús Tobal

**Affiliations:** 1 Department of Evolutionary Developmental and Educational Psychology, Campus Regional Excellence Mare Nostrum, University of Murcia, 30100 Murcia, Spain; cruiz@um.es (C.R.-E.); juanje.tobal@gmail.com (J.J.T.); 2 Department of Personality, Assessment and Psychological Treatment, Campus Regional Excellence Mare Nostrum, University of Murcia, 30100 Murcia, Spain

**Keywords:** CPRD, psychological variables, psychological profile, female soccer, competitive performance

## Abstract

The psychological variables that affect competitive performance are called the psychological profile of athletes. In recent years, the interest in female soccer players and the psychological characteristics that affect their performance has increased. The aim of the present study is to analyze the psychological characteristics of female professional soccer players and female amateur soccer players, as well as to determine the differences in the psychological profile of both groups. The participants were 134 federated female soccer players, with an average age of 18.28 years (SD = 4.05). To assess the psychological profile, the questionnaire on Psychological Characteristics related to Sports Performance (CPRD) by Gimeno, Buceta, and Pérez-Llantada (2001) was used. The results showed that female professional players presented higher values for motivation, while the female amateur players presented higher values for stress control and the influence of performance evaluation. These results can have a great impact on coaches’ work, since they can help them to establish tasks and training methods consistent with the characteristics of their players.

## 1. Introduction

During the last few decades, there has been a significant increase in the study of psychological characteristics related to sports performance [1]. The psychological variables that affect competitive performance are called the psychological profile of athletes [2,3]. This profile might be useful in training processes and can improve performances in competitions [4,5] due to the mediation that these psychological variables achieve between the physical, technical, and tactical abilities of the athlete and his/her performance [6,7]. Several authors have pointed out that attention control, self-confidence, coping strategies, and motivation are some of those variables [8,9]. Urra [10] found that training coping strategies were able to reduce precompetitive anxiety and improve performance during a competition. According to Días, Cruz, and Fonseca [11], self-confidence is a predictor of success in competitions.

Several investigations have shown that there is no single athlete profile. Instead, these profiles depend on other variables, such as the type of sport, gender of the athlete, and competitive level [12]. Some authors have studied the psychological profiles of athletes based on their competitive level. For example, Riddick [13] conducted a study on professional and amateur swimmers, indicating that amateurs manifest more positive personality traits than professional swimmers. More recently, López-Gullón et al. [9] pointed out the differences between amateur and professional athletes in Olympic and Greco-Roman wrestling, with self-confidence and attitudinal control differentiating them. Saber et al. [14] found differences in the profiles of expert and nonexpert taekwondo players. The studies by Vaca et al. [15] showed different levels of precompetitive anxiety in amateur, novice, and high-level karate competitors. In the field of soccer, Pestillo de Oliveira et al. [16] observed that professional players demonstrated more confidence and motivation to overcome the obstacles of the sports context than nonprofessional players. Other studies have highlighted gender differences in the psychological profiles of some athletes. Females showed higher levels of neuroticism, awareness, and kindness than males [17], as well as depression and anxiety [18]. Arias, Cardoso, Aguirre, and Arenas [19] have pointed out that while in female soccer, better values for team cohesion were registered, in volleyball, females scored higher in terms of motivation and mental ability. However, males have obtained higher values than females in aspects such as sports identification [20], self-esteem, ego, autonomy and enjoyment [21], and motivation [22]. Saber et al. [14] found different psychological profiles for male and female taekwondo players, and Alvarez et al. [23] found motivation and self-confidence as variables that change in the profiles, depending on gender. However, few studies have focused exclusively on the psychological profile of female athletes.

Soccer has been one of the sports more extensively studied in recent years in terms of the psychological profile of players, maybe due to the importance of these psychological variables in football training [12]. More recently, and due to a social change regarding the gender perspective, the interest in female soccer and the psychological characteristics that affect their performance has increased. Williams [24] has pointed out that the mental toughness, self-esteem, and anxiety control of female players are fundamental psychological traits for success. Arroyo del Bosque, Irazusta, and González-Rodríguez [25] focused on states of mood (tension, hostility, depression, vigor, and fatigue) of female soccer players. However, although there are other very interesting studies on female soccer [26,27], no studies have been found that compare the psychological profiles of amateur soccer players and those who are professionals. By knowing the psychological characteristics of professional players, the work of coaches and sports technicians, at all competition levels, could improve. Therefore, assessing the psychological capacities is a first step towards correct psychological training aimed at improving the performance of players, both professional and young, in training [28,29]. 

Therefore, the aim of this study was to analyze the psychological characteristics (stress control, influence of performance evaluation, motivation, and mental ability) of female professional soccer players and amateur soccer players, as well as to determine differences in the psychological profile of both groups.

The hypotheses of this study were as follows:

- Stress control will be higher in female professional soccer players than in amateur players;
- The influence of performance evaluation will be greater in female professional soccer players than in amateur players;
- Motivation will be higher in female professional soccer players than in amateur players;
- Mental ability will be higher in female professional soccer players than in amateur players;
- Team cohesion will be greater in female professional players than in amateur players.

## 2. Materials and Methods

### 2.1. Participants

A descriptive cross-sectional design was used. The participants were 134 female Spanish soccer players with a mean age of 18.28 years (SD = 4.05). Of these, 30.6% were professionals and the remaining 69.4% were amateurs. It was a nonprobability sample collected from the Soccer Association of the Region of Murcia (FFRM). The inclusion criteria were (a) being federated, (b) being a woman, (c) enrolling in a competition for at least three years, and (d) being older than 14 years. The only exclusion criterion was being injured. Of the 139 players who met the inclusion criteria, five were dismissed when applying the exclusion criteria. A professional player was defined as one who had a signed contract meaning that the player received financial compensation. All females who played in the second division and six females of the first division were considered as professionals (N = 41). The remaining players were listed as amateurs. Only 6.7% of the female players had competed in other sports; of these, 2.98% were professionals. Participation in the study was voluntary after the researchers reported on the study objectives. 

### 2.2. Instruments

Psychological variables were assessed using the Psychological Characteristics Related to Sport Performance Questionnaire (CPRD) [30] (see Table 1), based on the Psychological Skills Inventory for Sports (PSIS) [31]. The questionnaire consists of 55 items graded in a four-option Likert scale (from totally disagree to totally agree). 

CPRD includes five subscales: Stress control (SC), influence of performance evaluation (IPE), motivation (M), mental skills (MS), and team cohesion (TC). Cronbach’s alpha coefficient for the subscales is α_SC_ = 0.88, α_IPE_ = 0.72, α_M_ = 0.67, α_TCOH_ = 0.78 and α_MSK_ = 0.34, respectively. According to the authors, the low internal consistency of MS is probably related to this dimension including a wide range of different skills, but the subscale was kept within the study due to the factorials loads displayed by the items of this factor (over 0.30) [32]. 

Sociodemographic variables were assessed using an ad hoc questionnaire inserted at the beginning of the CPRD. The information collected is shown in Table 1.

### 2.3. Procedure

A list of females was requested from the Murcia Region Football Federation (FFRM). Subjects who met the sampling requirements for convenience were recruited. Later, an appointment was arranged with the coach, players, and their parents if they were minors. Finally, those players who agreed to participate in the research signed an informed consent form, and in the case of minors, their parents/guardians signed it. Tests were applied individually.

### 2.4. Data Analysis

First, a percentage analysis of the sociodemographic variables by both groups, professionals and amateurs, was carried out. Then, a descriptive analysis of each of the psychological variables (mean and standard deviation) was performed. Subsequently, a discriminant analysis [33] was conducted. Standardized coefficients greater than or equal to 0.30 [34] were considered relevant for the interpretation of the linear vectors. To ascertain the differences in each of the psychological variables independently, a Student’s *t*-test was used for independent samples.

The professionals and amateurs were compared by the method of magnitude-based inferences (MBI) (standardized Cohen’s d values and their 90% confidence intervals). The comparison of each psychological measure was done using Hopkins’ spreadsheet with the smallest worthwhile difference [35]. This method calculated 0.2 times the standardization, estimated from the between-subjects standard deviation. The magnitude of a clear difference was assessed as follows: >0.25%, trivial; 0.25%–75%, possibly; 75%–95%, likely; 95%–99%, very likely; >99%, most likely [36].

Statistical analysis was carried out through the statistical program IBM SPSS Statistics 23.0 (IBM Corp., Armonk, NY, USA), establishing the value of statistical significance at *p* < 0.05.

### 2.5. Ethical Approval

The study obtained the approval of the Ethical Committee of the University of Murcia (ID 2303/2019). This study was performed in accordance with the approved guidelines and the Declaration of Helsinki and with the informed consent of the participants.

## 3. Results

Several sociodemographic variables, such as educational level, player position, maximum division played, and federation years, have been analyzed (see Table 1). 

Table 2 shows the means and standard deviation of the different psychological variables under study in female professional and amateur soccer players.

Levene’s test did not show statistical differences. Therefore, the normality of the sample was assumed in all subscales, except for motivation: Stress control (*F* = 0.164, *p* = 0.686), influence of performance evaluation (*F* = 0.456, *p* = 0.500), motivation (*F* = 7.557, *p* = −0.007), mental ability (*F* = 3.381, *p* = 0.068), and team cohesion (*F* = 0.033, *p* = 0.866).

The data in Table 2 show higher values for motivation, mental ability, and team cohesion for female professional soccer players, although only motivation showed statistically significant differences. Moreover, female amateur soccer players exhibited higher values for stress control, and the influence of performance evaluation, with statistically related differences. The effect size (d) of the significant variables, stress control, influence of performance evaluation, and motivation, was medium or medium-low; therefore, these data must be taken with caution.

The results obtained from the discriminant analysis (Table 3) to determine the differences in the psychological profile of both groups demonstrated that the discriminant function was significant (*p* = 0.001) and correctly classified in 69.4% of the cases.

## 4. Discussion

The aim of this study was to analyses the psychological characteristics of professional female soccer players and amateur soccer players, as well as to determine the existing differences in the psychological profile of both groups. The results indicate that female professional soccer players present higher values for motivation, mental ability, and team cohesion, although only statistically significant differences in motivation were observed. On the other hand, the female amateur soccer players presented higher values for stress control and the influence of performance evaluation showed statistically significant differences.

As indicated, the female professional players displayed higher scores for motivation compared to the female amateur players. This subscale assesses the basic motivation for the sport practiced and the daily motivation related to the usual practices of their sport (training, lifestyle, travel, study-sport combination, etc.). Our results corroborate Pestillo de Oliveira et al.’s [16] study on male soccer players. In fact, being a professional may determine that players have a greater involvement with their sports practice and they live as if sports were very important for them. However, it is different in female amateur players, perhaps because they live in a more relaxing way than professionals. In addition, gender stereotypes may be affecting female motivation by pushing women to do something to demonstrate that they may do it as well as men do. In fact, females face stereotypes that have been generated for many years and face more pressure from society to play “male sports” than men [37].

However, the scores obtained in this study by female professional soccer players in the two factors of stress control and the influence of performance evaluation are lower than those obtained by amateur players. Amateur players may manage stress better than professionals because they are less affected by potentially stressful threats, and they manage the impact better due to the evaluation made by themselves or others of their performance. In this sense, in the study by Arias, Cardoso, Aguirre, and Arenas [19], male footballers showed superior scores for the influence of performance evaluation; that is, they better managed the impact of the evaluation. The greater responsibility perceived by the professional players with respect to their competency may be affecting the greater difficulties they face in stress management. In this sense, it is important to consider that soccer has historically been a sport practiced by men, with a very important masculine ideology burden [38,39], and the recent incorporation of women into it may have caused a sense of responsibility for what might be affecting their perception of stress. The perception of stress in sports practice, whether in training sessions or competitions, seems to be lived differently by males and females, and within females, differently by professionals and amateurs. In fact, it seems that the affected females perceive stressful situations as less controllable [40,41]. Therefore, it is possible that professional players will have a greater demand when evaluating their own performance and a greater sensitivity to the evaluations that others make about their performance.

Empirical evidence has shown that stress is a variable that can affect not only sports performance, but also other aspects, such as health (physical and psychological), well-being, and satisfaction with sports practice [42,43]. In this sense, the findings of this study have pointed out that, although the learning of psychological techniques and strategies may be fundamental for both professional and amateur players, it is much more necessary for professionals. These results are consistent with different studies on the role of psychological training programs [43,44].

Finally, the scores obtained in other subscales, such as team cohesion and mental ability, are higher in professional players, although these differences are not significant. It is possible that female professional players show a greater tendency to work in groups and strengthen the team by dedicating more hours and spending more time together in an effort to achieve a common goal [45,46]. This tendency is very important from the point of view of sports cohesion and cooperation, and its importance in performance and perceived subjective well-being is high [47,48]. As Basevitch [49] indicates, the psychological skills required to make optimal decisions and perform consistently in elite soccer include individual and team components that determine the interest in variables that influence team cohesion. In addition, one of the positive aspects involved from the perspective of team building is team cohesion, which can also be related to the perception of individual performance. According to Olmedilla et al. [27], who carried out a study on sub-16 and sub-18 soccer players, team cohesion is negatively related to the player’s perception of performance. It may be that individual exposure to performance evaluation is experienced with a high stress load, and “refuge is sought” in the cohesion of the sports team. Otherwise, it is also possible that female professional players have more psychological resources to face sports practice than amateurs. It seems necessary to improve the use of techniques and strategies that increase their psychological skills and abilities, and thus are able to adequately manage stress and the impact of evaluations of their performance [50,51,52,53]. In addition, gender seems relevant when addressing psychological training [54], given that both the repertoire of psychological skills (relaxation, negative thinking in competitions, internal dialogue during competitions, and visualization) and interest in this type of work are not equal for females and males. Moreover, these results are of great importance for the coach’s work, since it can help them establish tasks and training methods that are more in line with the characteristics of their players, whether professional or amateur [55,56,57].

These findings should be treated with caution due to a number of limitations. First, this investigation includes a convenient sample, and studies should be carried out on a larger population. Secondly, the study design is cross-sectional, so the absence of repeated measures must be taken into account. Longitudinal studies that corroborate these findings are required. Finally, this study has not taken into account the age variable. Although there is no consensus on the influence of the age variable on certain psychological characteristics, future research is necessary.

## 5. Conclusions

The main conclusions of the present study, with the sample referred, are as follows:

- Female professional soccer players show a higher level of motivation than female amateur soccer players, in terms of both basic motivation for practicing sports and daily motivation related to the usual practices of their sport.
- Female amateur soccer players show better stress management and better management of the impact of performance evaluation than female professional soccer players.

## Figures and Tables

**Table 1:** Sociodemographic data of professionals and amateurs.

	**Totals**		**Professional**		**Amateurs**	
**Educative Level**	**N**	**Percentage (%)**	**N**	**Percentage (%)**	**N**	**Percentage (%)**
Compulsory Secondary Education	72	53.7	13	9.7	59	44.0
Bachelor	55	41.0	26	19.4	29	21.6
Vocational Education	7	5.2	2	1.5	5	3.8
Total	134	100	41	30.6	93	69.4
**Soccer Player Position**	**N**	**Percentage (%)**	**N**	**Percentage (%)**	**N**	**Percentage (%)**
Goalkeeper	15	11.2	2	1.49	13	9.7
Defender	45	33.6	10	7.46	35	26.1
Midfielder	42	31.2	17	12.6	25	18.6
Forward	32	23.9	12	8.9	20	14.9
Total	134	100	41	30.6	93	69.4
**Maximum Division Played**	**N**	**Percentage (%)**	**N**	**Percentage (%)**	**N**	**Percentage (%)**
Second division league	35	26.1	35	26.1	0	0
Third division league	83	61.9	0	0	83	61.9
First division league	6	4.5	6	4.5	0	0
Regional league	8	6.0	0	0	8	6.0
Local league	2	1.5	0	0	2	1.5
Total	134	100	41	30.6	93	69.4
**Soccer Federation Years**	**N**	**Percentage (%)**	**N**	**Percentage (%)**	**N**	**Percentage (%)**
<4 years	43	32.0	9	6.7	34	25.3
From 4 to 9 years	51	38.2	19	14.2	32	24
>9 years	40	29.8	13	9.7	27	20.1
Total	134	100	41	30.6	93	69.4

**Table 2:** Scores of the psychological variables under study (mean, standard deviation, and significance level) of female professional and amateur soccer players.

Psychological Factors	Professional	Amateur	*p*	d
Stress control	27.22 ± 11.54	34.32 ± 12.36	0.002	0.59
Influence of performance evaluation	18.27 ± 6.13	20.28 ± 6.72	0.050	0.31
Motivation	13.39 ± 5.04	11.85 ± 3.24	0.036	0.36
Mental skills	15.29 ± 4.47	14.24 ± 3.36	0.134	0.26
Team cohesion	6.24 ± 4.12	6.19 ± 2.73	0.934	0.01

**Table 3:** Standardized coefficients of the discriminant analysis between professional and amateur players.

Psychological Factors	Standardized Coefficients
Stress control	0.637 *
Influence of performance evaluation	0.399 *
Motivation	−0.432 *
Mental skills	−0.307 *
Team cohesion	−0.017

* SC discriminant value ≥ 0.30.
